# In-vitro and in-vivo metabolism of different aspirin formulations studied by a validated liquid chromatography tandem mass spectrometry method

**DOI:** 10.1038/s41598-021-89671-w

**Published:** 2021-05-14

**Authors:** Michele Dei Cas, Jessica Rizzo, Mariangela Scavone, Eti Femia, Gian Marco Podda, Elena Bossi, Monica Bignotto, Sabrina Caberlon, Marco Cattaneo, Rita Paroni

**Affiliations:** 1 Laboratory of Clinical Chemistry and Mass Spectrometry, Department of Health Sciences, Università degli Studi di Milano, ASST-Santi Paolo e Carlo, via di Rudini′ 8, 20142 Milan, Italy; 2 Laboratory of Hemostasis and Thrombosis, Department of Health Sciences, Università degli Studi di Milano, Milan, Italy; 3 Medicina III, ASST-Santi Paolo e Carlo, Milan, Italy; 4 Internal Medicine and Liver Unit, Department of Health Sciences, Universita′ degli Studi di Milano, Milan, Italy

**Keywords:** Vascular diseases, Thromboembolism, Thrombosis, Medical research

## Abstract

Low-dose aspirin (ASA) is used to prevent cardiovascular events. The most commonly used formulation is enteric-coated ASA (EC-ASA) that may be absorbed more slowly and less efficiently in some patients. To uncover these “non-responders” patients, the availability of proper analytical methods is pivotal in order to study the pharmacodynamics, the pharmacokinetics and the metabolic fate of ASA. We validated a high-throughput, isocratic reversed-phase, negative MRM, LC–MS/MS method useful for measuring circulating ASA and salicylic acid (SA) in blood and plasma. ASA-d4 and SA-d4 were used as internal standards. The method was applied to evaluate: (a) the "in vitro" ASA degradation by esterases in whole blood and plasma, as a function of time and concentration; (b) the "in vivo*"* kinetics of ASA and SA after 7 days of oral administration of EC-ASA or plain-ASA (100 mg) in healthy volunteers (three men and three women, 37–63 years). Parameters of esterases activity were *V*_*max*_ 6.5 ± 1.9 and *K*_*m*_ 147.5 ± 64.4 in plasma, and *V*_*max*_ 108.1 ± 20.8 and *K*_*m*_ 803.2 ± 170.7 in whole blood. After oral administration of the two formulations, *t*_max_ varied between 3 and 6 h for EC-ASA and between 0.5 and 1.0 h for plain-ASA. Higher between-subjects variability was seen after EC-ASA, and one subject had a delayed absorption over eight hours. Plasma AUC was 725.5 (89.8–1222) for EC-ASA, and 823.1(624–1196) ng h/mL (median, 25–75% CI) for plain ASA. After the weekly treatment, serum levels of TxB_2_ were very low (< 10 ng/mL at 24 h from the drug intake) in all the studied subjects, regardless of the formulation or the *t*_max_. This method proved to be suitable for studies on aspirin responsiveness.

## Introduction

Low-dose acetylsalicylic acid (ASA, 75–100 mg/o.d.) is used in clinical practice to reduce the risk of arterial thrombosis^1^. ASA irreversibly inactivates platelet cyclooxygenase-1 (COX-1) enzyme, thus preventing the formation of the platelet agonist thromboxane A_2_ (TxA_2_)^2^. Measurement in serum of TxB_2_ , the stable TxA_2_ metabolite, is used to evaluate the pharmacologic efficacy of ASA^3^ which is able to induce a decrease of TxB2 levels from 200 to 300 ng/mL to ≤ 10 ng/mL^4,5^ when administered in healthy subjects. Enteric-coated aspirin (EC-ASA) is commonly used in clinical practice because it was initially hypothesized that it would cause less gastrointestinal discomfort and bleeding^6–8^, compared to plain ASA. This surmise was not confirmed by controlled studies^9^, but EC-ASA is still the most commonly used ASA formulation to prevent arterial thrombosis. In recent years, it has been shown that, despite its extensive use, a significant proportion of patients taking the therapeutic dose of EC-ASA (100 mg o.d) displays inadequate pharmacodynamics (PD) response, with partial or null inhibition of TxA_2_biosynthesis or of arachidonic acid-induced platelet aggregation, thus increasing the probability of atherothrombotic and ischaemic events. Although "resistance" to ASA in patients with coronary artery or cerebrovascular disease is relatively rare^10^, conversely it is rather frequent in some categories of patients at high risk of thrombotic events, such as those undergoing coronary artery bypass or those with essential thrombocytemia (TE)^1,11^. A recent meta-analysis reported that the overall prevalence of "laboratory defined" aspirin resistance in CVD patients is 24.7%^12^, and although nowadays a standardized test that could identify “resistant patients” is lacking, it’s clear that subjects on ASA therapy show a great variability in response to ASA. These patients are classified as "non-responders", or having an absent or incomplete pharmacological response to therapy^3^, for reasons not yet well clarified.

The potential mechanisms for “ASA resistance” has been largely investigated^13–15^ and may be caused by several factors: (a) poor adherence; (b) decreased efficiency of some formulations of ASA to inhibit TxA_2_ production^16–19^; (c) inadequacy of the standard low doses of daily aspirin (81–100 mg) to inhibit completely COX-1 activity^5,20,21^ (to these patients, less protected from thrombotic events, treatment with 100 mg b.i.d. regimen or with a different ASA formulation has been proposed^16–19^); (d) competition of ASA with other NSAIDs (for example ibuprofene, indometacine) which could block ASA access at binding site (Ser-530) in COX-1^22^; (e) unknown interaction with proton pump inhibitors (PPIs) routinely co-prescribed to patients on ASA chronic treatment at high risk of bleeding; (f) esterase-mediated metabolism of ASA: in vivo ASA may undergo hydrolysis to salicylate prior to absorption because of esterases in the gastrointestinal tract^23^. Variations in blood-borne esterase activity have been documented in healthy subjects (HS)^24^, but attempts to correlate activity with pathological states have yielded inconsistent results; (g) platelet multidrug resistance protein-4 (MRP4) overexpression^25,26^; (h) increased production of platelets with a new COX-1 not inhibited by ASA and able to synthetized TxA_2_^10^; (i) genetic polymorfirsm: presence of COX-1 variants that may be less responsive to aspirin inhibition^27,28^ (l) biosynthesis of TxA_2_ by pathways that are not blocked by ASA; (m) interventions of coronary revascularization with coronary artery bypass surgery or coronary angioplasty that may induce temporary aspirin resistance^10^; (n) loss of antiplatelet effect of ASA with prolonged administration: tachyphylaxis.

Among all these different assumptions, the most plausible are (a) the lack of a pharmacodynamics effect; (b) the presence of excessive esterase activity; (c) the presence of an impaired intestinal absorption. To verify these assumptions, it is necessary to evaluate «in vitro» the esterases activity in plasma and blood, and to study «in vivo» the kinetics of ASA and salicylic acid (SA), and TxB2 inhibition.

While different commercial methods are available for TxB2 evaluation, ASA and SA detection needs a fully validated method based on isotope dilution liquid chromatography–mass spectrometry (LC–MS/MS). Consequently, the availability of a suitable analytical procedure is pivotal for any clinical study on aspirin responsiveness.

Here we describe the set-up and optimization of the LC–MS/MS method, and we demonstrate its suitability for further clinical studies by assessing: (1) the "in vitro" ASA-degrading esterase activity in whole blood and plasma of a small cohort of HS; (2) the "in vivo" ASA and SA plasma pharmacokinetics after oral administration of two different ASA formulations (EC-ASA and plain ASA) to HS. To complete our pilot study with pharmacodynamics information, serum TxB_2_ was also measured.

## Results

### LC–MS/MS method for aspirin quantification in plasma

**Figure 1:**
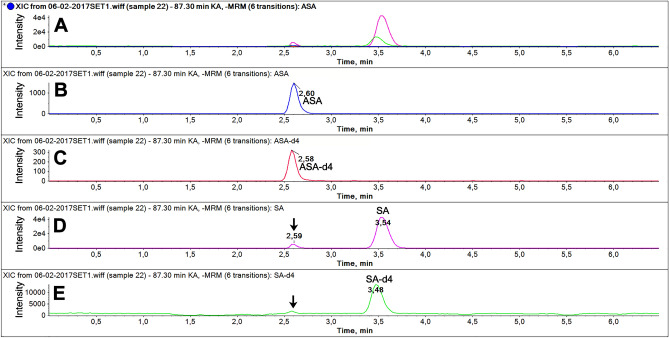
(**A**) extracted ion (XIC) chromatograms of all components in a plasma sample; (**B**) XIC of ASA; (**C**) XIC of ASA-d4; (**D**) XIC of SA; (**E**) XIC of SA-d4. Arrows indicate the chromatographic peaks of SA in-source generated from ASA and ASA-d4, respectively.

Optimization of mass spectrometry and liquid chromatography conditions, fragment ion spectra of ASA and SA, and the Compounds Parameter for each analyte are fully described in “Supplemental Data” (Note S1, Figs. S1, S2, and Table S1). It is worthy to note that more than 50% of ASA undergoes in-source fragmentation and forms SA, likewise ASA-d4 forms SA-d4. Liquid chromatography plays an important role in the method development of ASA and SA: in pharmacokinetics (PK) studies, chromatographic separation is pivotal to distinguish between the SA fragment peak generated into the source, and the SA generated in vivo during ASA metabolism. Figure 1 reports an example of chromatograms of all components: retention times were 2.6 min for ASA and ASA-d4; 3.5 min for SA and SA-d4.

### Method validation

The developed method was validated according to the FDA and EMA guidance for bioanalytical method validation^29,30^ and performed using MultiQuant software 2.1 and GraphPad Prism v. 7.0. The parameters determined were: selectivity, specificity, linearity, precision, accuracy, recovery and stability.

Selectivity was determined by analysing six blank plasma samples, obtained from six different sources, spiked with analytes and internal standards at the respective lowest limit of quantification (LLOQ) concentration.

As reported in FDA guidelines^29^, the lowest standard on the calibration curve should be accepted as LLOQ if the analyte response is at least five times the response compared to blank (solvent) response and analyte peak should be identifiable, discrete and reproducible with a precision of ± 20% and accuracy of 80–120%^29^. Using this analytical method LLOQ for ASA and SA was 20 ng/mL. The limit of detection (LOD) was determined considering the lowest concentration at which signal to noise ratios was > 3^30^. For ASA and SA LOD was 10 ng/mL. Figure S3 reports the chromatograms of blank plasma samples and Fig. S4 the chromatograms of ASA and SA at the LLOQ level.

The linearity of the method was evaluated with analyte-spiked plasma samples by the use of   the least squares method. Calibration curves were linear over the concentration range of 20–2000 ng/mL [y = 0.003621 (± 0.000043)x + 0.006301 (± 0.03325); R^2^ = 0.9991] for ASA and 20–8000 ng/mL [y = 0.001937 (± 0.000004)x + 0.01326 (± 0.01249) R^2^ 0.9999] for SA.

Intra- and inter-day accuracy and precision were evaluated by the analysis of six replicates containing ASA and SA at different QC levels (20, 60, 400 and 1250 ng/mL for ASA; 20, 60, 200, 500 and 5000 ng/mL for SA) prepared on the same day and in different days. The accuracy was expressed as % bias: (observed concentration)/(nominal concentration) × 100 and precision by % of the CV. The acceptable criteria of the data included accuracy within ± 15% deviation from the nominal value and precision within ± 15% CV except for LLOQ, which was set at ± 20%. Results of intra-day and inter-day accuracy and precision are reported in “Supplemental Data”, Tables S2–S3. Intra-day and inter-day precision were within ± 15% for each QC at low medium and high levels and within ± 20% at LLOQ levels. The intra-day and inter-day accuracies were all within 100 ± 15% of the nominal value and were within 100 ± 20% at LLOQ levels.

Proteins precipitation with acetonitrile containing 0.1% of formic acid was trustworthy and provided clean samples. The recoveries of analytes were good and reproducible: for ASA at concentrations of 20, 100, 500 and 2000 ng/mL, recoveries were 85.60 ± 4.74, 68.46 ± 0.67, 57.12 ± 4.15 and 56.02 ± 5.05; for SA at concentrations of 20, 100, 500, 5000 and 8000, they were 72.93 ± 5.0470, 71 ± 0.80, 77.76 ± 4.16, 77.85 ± 0.61 and 71.39 ± 2.06. Internal Standard  normalized matrix factor was calculated as reported by Sirok et al.^31^ and the CV% values were 13.1%, 8.26%, 2.83%, 2.20% at ASA concentration of 20, 100, 500, 2000 ng/mL and 5.02%, 4.30%, 4.34%, 5.21% and 4.08% at SA concentration of 20, 100, 500, 5000 and 8000 ng/mL.

Stability experiments of ASA and SA in plasma samples were carried out by analysing QC samples at two different concentrations for ASA (60, 1250 ng/mL) and three different concentrations for SA (60, 200 and 5000 ng/mL), under three different conditions: after three freeze–thaw cycles (− 20 °C; 5 °C), after short term storage (6 h) in ice-bath, after long-term stability (2 months at − 20 °C). Short term stability of post-extracted plasma was also evaluated in autosampler at 5 °C for 72 h. No significant degradation of ASA and SA was observed under the conditions studied (Tables S4, S5).

### Esterase activity in plasma and blood

**Figure 2:**
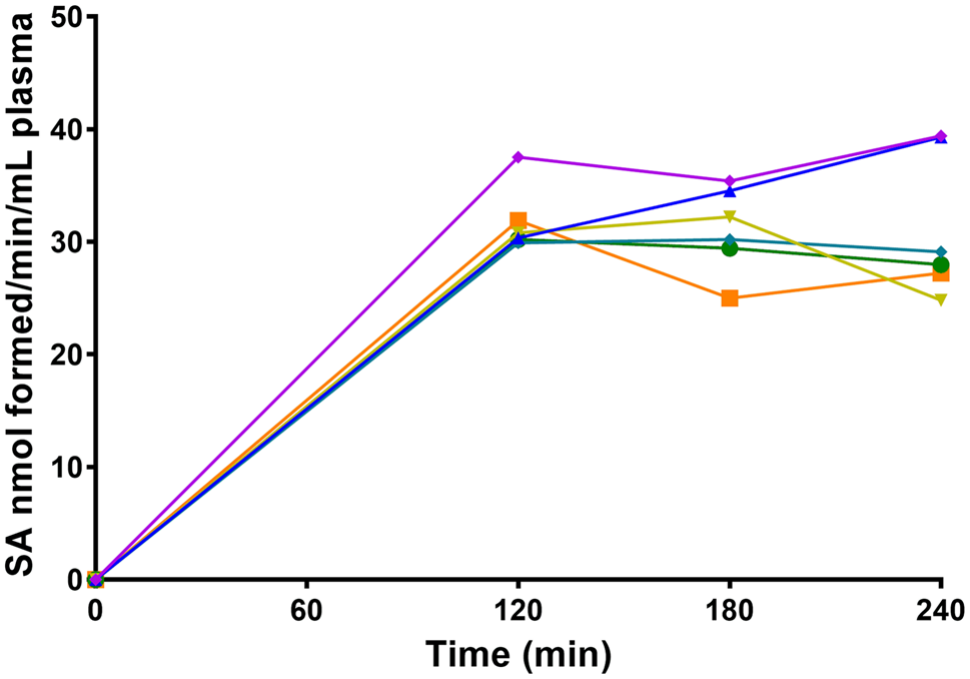
Esterase activity (SA formed) as a function of time in HS (n = 6) incubating ASA 40 mM as a substrate.

The LC–MS/MS method was applied to study the "in vitro" plasma esterase activity in the study subjects. Figure 2 shows the plasma enzyme activity as a function of time. Maximal activity was observed after 120 min of incubation, then declined.

**Figure 3:**
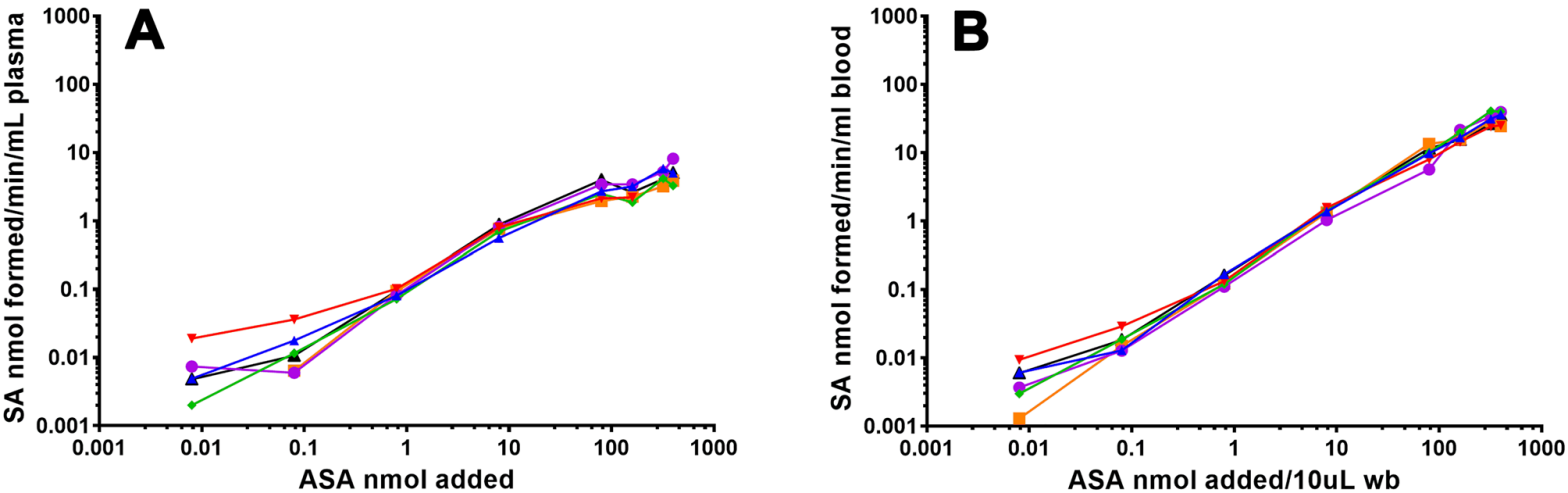
Esterase activity after 2 h incubation as a function of substrate concentration in plasma (**A**) and whole blood (**B**).

Esterase activity, as a function of substrate concentration, was studied both in whole blood and in plasma (Fig. 3). Using the Michaelis–Menten model, V_max_ and K_m_ were 6.54 ± 1.87 nmol/mL/min and 147.5 ± 64.4 nmol/10 µL in plasma, and 109 ± 20.8 nmol/ mL/ min and 803 ± 171  nmol/10 µL (mean ± SEM, n = 10), in whole blood.

### Pharmacokinetics of ASA formulations in healthy subjects

**Figure 4:**
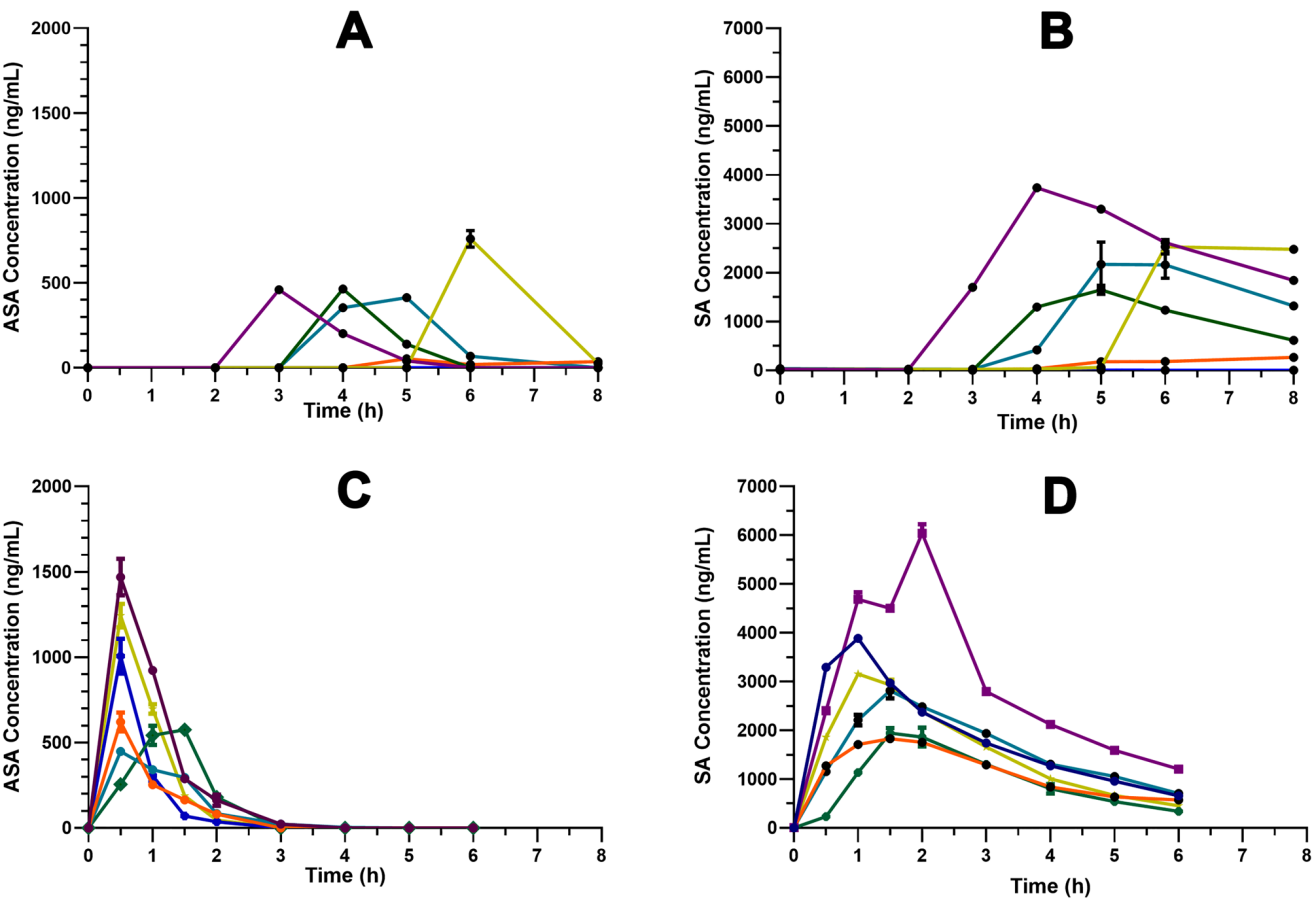
Plasma levels of ASA and SA in HS (n = 6) after EC-ASA and plain-ASA administration. In detail: (**A**) ASA levels after EC-ASA treatment; (**B**) SA levels after EC-ASA treatment; (**C**) ASA levels after plain-ASA treatment and (**D**) SA levels after plain-ASA treatment.

Figure 4 shows the plasma concentration–time profile of ASA and SA in each HS studied  (n = 6) after EC-ASA and plain-ASA intake. At each time point, ASA and SA were recorded simultaneously in plasma.

**Figure 5:**
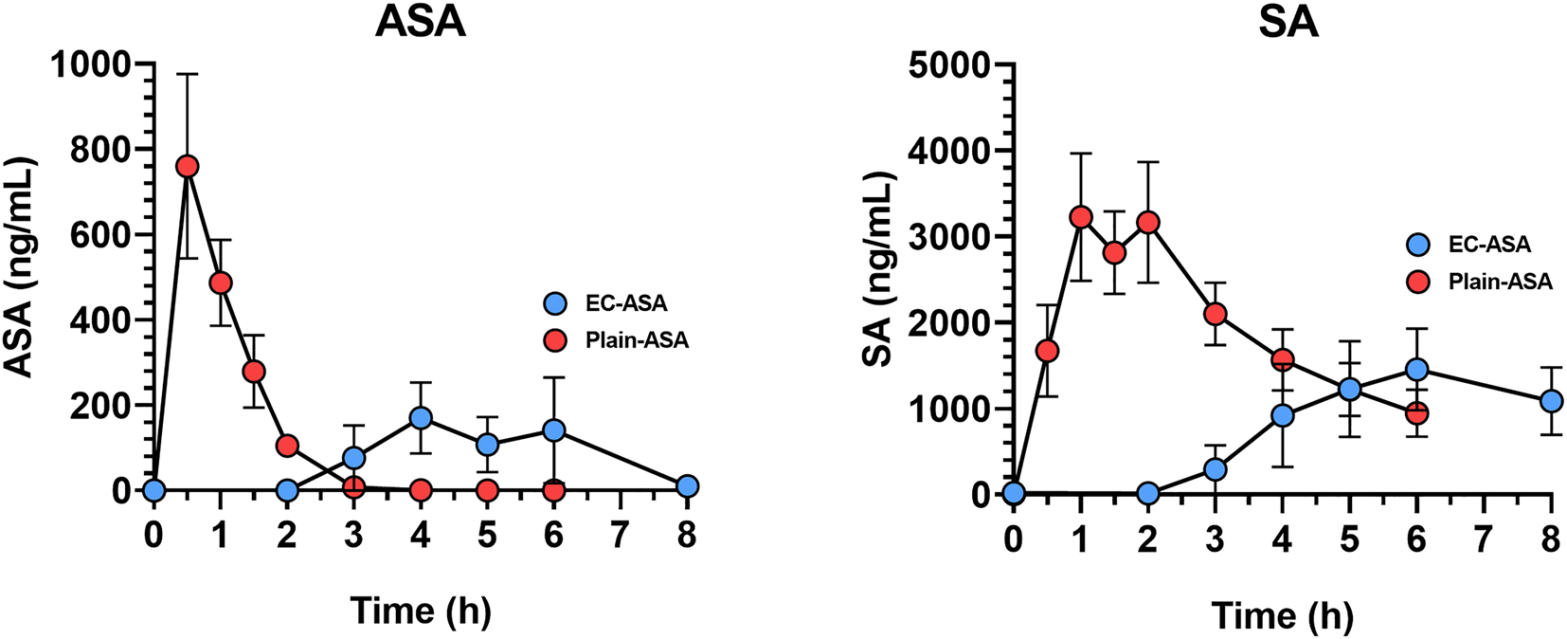
Mean pharmacokinetics curves after oral administration of 100 mg EC-ASA (blue) and plain-ASA (red) to HS (n = 6). The curve after EC-ASA administration includes the subject with no absorption.

**Figure 6:**
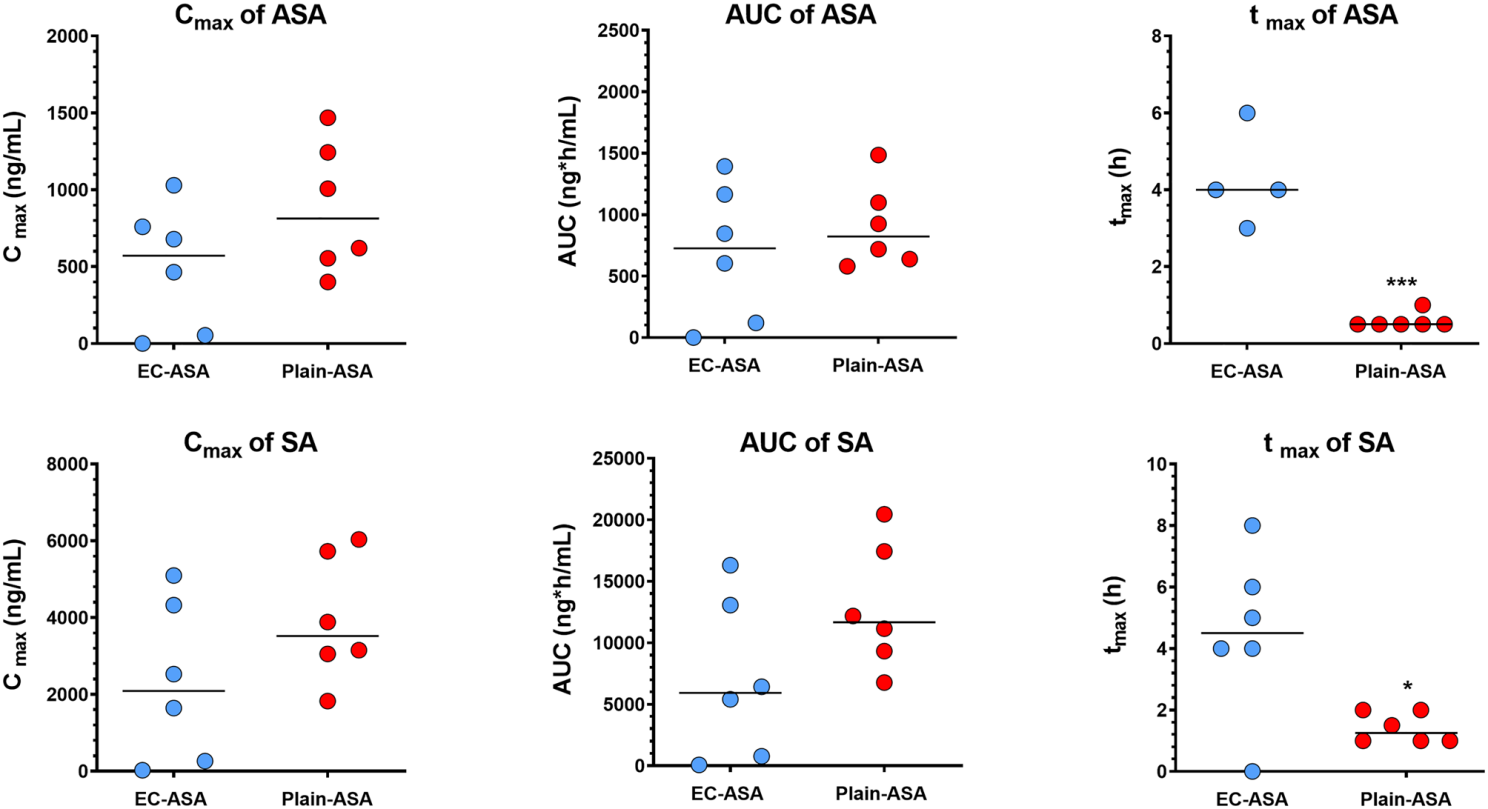
PK parameters of ASA (above) and SA (below) in 6 HS after EC-ASA (blue) and plain-ASA (red) administration. Only T_max_ of ASA and SA were significantly different between the two treatments. To note that t_max_ after EC-ASA administration could be calculated only on n = 4 patients.

After EC-ASA administration, the drug reached the plasma compartment quite variably among subjects, and never before 2 h from its administration. No ASA or SA signal was observed in plasma from one subject across the 8 h of observation (Fig. 4). The mean of time-concentration pharmacokinetics curves for ASA and SA are reported in Fig. 5. The median maximal ASA plasma concentration (C_max_), which was observed between 3 and 6 h, was 571.7 (IQR, 40.28–827.1) ng/mL, and the median AUC was 725.5 (89.8–1222) ng h/mL. SA C_max_ and AUC were 2089 (206–4518) ng/mL and 5920 (600–13,883) ng h/mL, respectively (Fig. 6).

After administration of plain ASA, drug absorption occurred in all subjects between 0.5 and 1.5 h after intake. ASA C_max_ and AUC were 814 (516–1300) ng/mL and 823 (624–1196) ng h/mL; SA C_max_ and SA AUC were 3520 (2748–5805) ng/mL and 11,663 (8648–18,194) ng h/mL (median, IQR) (Figs. 4 and 5). By comparing the PK parameters between the two ASA formulations, only *t*_max_ was statistically different both for ASA (p < 0.0001) and SA (p = 0.0002).

The pharmacodynamics of the two tested ASA formulations was evaluated by measuring the serum levels of TxB_2_. Its concentrations were very low (< 10 ng/mL at 24 h from drug intake) in all the subjects after 7 days of daily treatment with 100 mg ASA, regardless of the formulation.

## Discussion

Availability of a robust and validated analytical procedure is essential for developing clinical protocols aimed at studying in vivo ASA metabolism.

Several published methods are currently available for the simultaneous quantification of ASA and its metabolite. Sirok et al.^31^ recently published a sensitive method for determining ASA and SA using ASA-d4 and SA-d4 as internal standards, a liquid–liquid extraction followed by an evaporation step and chromatographic gradient separation. The method allowed to obtain LLOQs of 1 ng/mL for ASA and 80 ng/mL for SA. Xu et al.^32^ extracted ASA and SA from human plasma using protein precipitation with 6-methoxysalicylic acid as internal standard and an isocratic chromatographic separation. Barathi et al.^33^ get a LLOQ of 1.09 ng/mL and a relatively rapid chromatographic separation but included an SPE extraction procedure. Chhonker et al.^34^ proposed the simultaneous quantitation of ASA and clopidogrel along with their metabolites. The pre-analytical purification included a precipitation step, a liquid–liquid extraction and a drying step. This method reached a LLOQ of 1.56 ng/mL for both ASA and SA.

With the perspective of using the analytical method to study PK of ASA in large clinical trials on patients under chronic treatment, and/or to compare different drug formulations, none of the described methods met our needs, so we elected to develop a rapid, sensitive, robust and cheap method. Despite the desired cheapness and robustness of analysis, aspirin quantification for clinical studies also needs great accuracy. Due to the known in-source aspirin degradation, this can be ensured by a proper chromatographic resolution of ASA and SA peaks and by the use of stable-labelled internal standards, highly recommended for each analytical method based on LC–MS/MS. We propose an isocratic mobile phase that allows high throughput analysis and avoids the tedious long re-equilibration time that is necessary in gradient analysis and, in the meanwhile, we get the baseline separation (~ 1 min) of ASA and SA within 6 min with good S/N ratio (Fig. S4). The method was linear over a large concentrations range as necessary in PK studies, and LLOQ was 20 ng/mL for both analytes. We chose simple protein precipitation with organic solvent avoiding longer and more time-consuming procedures. Of course, we are aware that this would result in a sensibility lower than the ones reported by others, but, if necessary, those lower limits can also be yielded with our method by merely drying the sample and dissolving with a smaller volume of the solvent prior LC–MS/MS analysis.

In vivo, ASA undergoes spontaneous and enzymatic hydrolysis by erythrocyte and plasma esterase. In plasma, enzymatic activity is mainly corresponding to that of plasma cholinesterase, while a minor activity is due to albumin acting as an esterase. In whole blood esterase activity is greater than in plasma, and an arylesterase with specificity for aspirin has been isolated from human erythrocytes^35^. Zhou et al.^36^ showed that ASA is firstly hydrolysed within erythrocytes by PAF acetylhydrolase; however, this enzyme activity has been verified to be very variable among individuals. In a subsequent study, Zhou et al.^37^ showed a statistically significant higher plasma esterase activity in 1928 coronary artery disease patients (16.5 ± 4.4 nmol/mL/min) compared with 298 control subjects (15.1 ± 3.7 nmol/mL/min) (p = 3.4 × 10^–8^). Excessive esterase metabolism could cause the loss of PD effect of ASA treatment observed in some patients. Here, using plasma of HS and LC–MS/MS, we set-up a precise protocol to study esterase activity in both whole blood and plasma. This was necessary to rule out the contribution of the plasma activity from that of leucocytes and erythrocyte. This method is therefore suitable to investigate cohorts of patients at risk of inadequate response to ASA.

The LC–MS/MS method was also applied to study PK of two different low-dose ASA formulations in human plasma. ASA is rapidly and completely absorbed from the gastrointestinal tract. During and after absorption, ASA is converted to its main metabolite SA. Plain-ASA is rapidly absorbed in the stomach, while the enteric-coating of EC-ASA is resistant to the acid environment and, therefore, it releases ASA for absorption in the alkaline environment of the intestine, rather than in the stomach. From our results, it appears clear that in subjects on treatment with EC-ASA, ASA and SA in plasma peak from 2 to 6 h, with significant inter-individual variability. After witnessed intake of EC-ASA, one subject showed a flat time-concentration serum line for both ASA and SA during all 8 h of observation. This was probably due to an impaired dissolution of the enteric coating in the alkaline environment of the intestine, preventing the release of the active ingredient, or to an impaired intestinal function of the subject, causing a delayed intestinal absorption across the cells membrane. This hypothesis may be plausible as the EC-ASA pharmacodynamics effect was also demonstrated in this patient, with TxB2 concentrations very low (< 10 ng/mL) at 24 h from the drug intake.

On the contrary, plain-ASA was rapidly absorbed with a t_*max*_ between 0.5 and 1.5 h, with minor inter-individual variability. Despite the reported differences in the absorption time, and excluding the subjects with impaired absorption, the two treatments were comparable in plasma AUCs. Notably, all the patients had inhibition of TxB_2_ formation also after 24 h from the drug intake (< 10 ng/mL)^36^.

## Conclusion

Our procedure allows studying the analytical fate of ASA "in vivo" following plasma ASA and SA levels. We compared two different ASA formulations: the enteric-coated formulation showed an irregular behaviour and absorption, which was impaired in one subjects. On the contrary, plain-ASA absorption variability was lower, as all tested subjects absorbed the drug efficiently. Our procedures could be used for studying ASA metabolism in patients who display inadequate response to the drug.

## Materials and methods

### Materials

ASA, SA, HPLC-grade acetonitrile, formic acid, potassium fluoride were purchased from Sigma Aldrich (now Merck KGaA, Darmstadt, Germany). Acetylsalicylic acid-d4 (ASA-d4), salicylic acid-d4 (SA-d4) were purchased from Santa Cruz Biotechnology (CA, USA). Inertsil ODS3, 150 × 3.0 mm i.d., 3 μm (GL Sciences, Tokyo, Japan) was used for chromatography separation. Ultrapure water was obtained from MilliQ system. TxB_2_ EIA kit was purchased from Cayman Chemicals (Ann Arbor, MI, USA). EDTA KE/2.7 mL, coagulation 9 NC/3 mL, and serum Z/2.7 mL S-Monovette were purchased from Sarstedt (Verona, Italy). Plain-ASA and EC-ASA tablets at a dose of 100 mg were purchased from Bayer (Leverkusen, Germany).

### LC–MS/MS instrumentation

The mass spectrometry measurements were performed with the same instrument previously described^38^. Briefly, the liquid chromatography system was an UltiMate 3000 LC Systems (Dionex, Sunnyvale, CA, USA), with autosampler, binary pump, and column oven (Thermo Fisher Scientific, USA). The tandem mass spectrometer was an AB Sciex 3200 QTRAP LC–MS/MS instrument with electrospray ionization (ESI) TurboIonSpray source (AB Sciex Framingham, MA, USA). Instruments were managed with manufacturers' software and according to manufacturers' instructions. The analytical data were processed by Analyst software (version 1.6.2).

### Quantification of ASA and SA in human plasma

The isotope dilution LC–MS/MS (ID-LC–MS–MS) technique in negative multiple reaction monitoring mode (MRM) was developed for the determination of ASA and SA using the respective deuterated isotopomers (ASA-d4 and SA-d4) as internal standards (IS). In order to optimize ESI conditions for ASA, ASA-d4, SA and SA-d4, full scans were carried out in negative ion detection mode by infusing each analyte at a concentration of 10 µg/mL. The most abundant product ions were chosen in the MRM acquisition in terms of better sensitivity: for ASA and SA at m/z 179.0 > 137.0 and m/z 137.0 > 93.0; for ASA-d4 and SA-d4 at m/z 183.0 > 141.0 and m/z 141.0 > 97.0, respectively. During the infusion experiments, source parameters and compound parameters were also optimized (“Supplemental Data”, Note S1, Figs. S1, S2). ASA, ASA-d4, SA and SA-d4 were separated on reversed-phase column Inertsil ODS3, 150 × 3.0 mm i.d., 3 μm particle size with an isocratic elution by mobile phases consisting of acetonitrile and water containing 0.1% formic acid (80:20, v/v).

### Preparation of stocks, calibrators, and quality controls

Two independent calibration curves were prepared in human plasma (250 µL, containing KF 1 mg/mL) by spiking 50 µL of the appropriate working solution giving a final concentration of 20, 50, 100, 200, 500, 1000, 2000 ng/mL for ASA and 20, 50, 100, 200, 500, 1000, 2000, 5000, 8000 ng/mL for SA. A specific SA calibration curve between 0.1 μM and 0.2 mM was constructed to study esterases activity.

Quality control (QC) samples were prepared by spiking control human plasma in bulk with ASA and SA at appropriate concentrations in the low, medium and high range: for ASA 20, 60, 500 and 1500 ng/mL; for SA 20, 100, 500, 5000 and 8000 ng/mL. The developed method was validated according to the FDA and EMA guidance for bioanalytical method validation^29,30^.

### Sample preparation for mass spectrometry

ASA and SA were purified from human plasma using protein precipitation: 250 µL of human plasma were added 25 µL of ASA-d4 (4 µg/mL), 25 µL of SA-d4 (10 µg/mL) and 700 µL of 0.1% formic acid in acetonitrile. Samples were processed in an ice-bath in order to prevent ASA hydrolysis by esterase. The mixture was vortexed for 1 min, then centrifuged at 14,000*g*, 4 °C for 10 min. The supernatant was transferred into an analytical vial, and 10 µL were injected into the LC–MS/MS system.

### Study population

Six HS were recruited among clinicians and students at the Department of Health Sciences. They included three women and three men subjects aged 53 (37–63) yrs and with BMI 22.7 (19.4–27.2); median (CI 25–75%). For haematological data 3 mL blood were collected into K-EDTA tubes and analysed by Coulter analyser. Blood count values were WBC (× 10^9^/L) 6.1 (5.4–6.8); RBC (× 10^12^/L) 4.8 (4.3–5.0); Hb (g/dL) 14.3 (13.4–15.4) Haematocrit (%) 41.1 (39.2–43.0); Platelets (× 10^9/^L) 223 (196–242): MPV (fL) 7.7 (7.4–7.9), (median; CI 25–75%).

All subjects, who voluntarily accepted to participate in the study, were informed and authorization was obtained by signing a letter of consent. These subjects were chosen among those who participated to a larger clinical study^1^ approved by the institutional local ethical committee (Comitato Etico, Ospedale San Paolo, Milano, Italy). None of the volunteers was under pharmacological treatment. The exclusion criteria were: pregnancy, lactation and nonsteroidal anti-inflammatory drug assumption. A diary containing information about drugs assumption, weight, height, breakfast, age, and withdrawal times was written off for each enrolled subject.

### Esterase activity in plasma and blood

For measuring esterase activity in plasma and whole blood, 3 mL of blood were collected from HS in commercial citrate tubes: blood was immediately aliquoted and processed, while, after centrifugation at 1400*g* for 15 min, plasma was separated and stored at − 80 °C.

Esterase activity was studied by incubating plasma samples in the presence of ASA as a substrate, and following the SA formation as a function of both time and substrate concentration. Plasma esterase activity as a function of time was studied following the experimental condition described by Zhou et al.^37^. In brief, 10 μL of plasma were added with 40 μL of ASA (4 mM) and incubated at 37 °C for 120, 180 and 240 min, before stopping the reaction by 150 μL of acetonitrile containing 0.1% of formic acid. Enzyme activity as a function of substrate concentration was studied both in plasma and in whole blood: 10 μL were added with 40 μL of ASA (from 0.2 μM to 10 mM, 0.008–400 nmol added) and incubated at 37 °C for 120 min, then the reaction was stopped by 150 μL of acetonitrile containing 0.1% of formic acid. Samples were centrifuged at 14,000*g* for 10 min and opportunely diluted (between 1:10 and 1:375) before LC–MS/MS analysis. To 100 μL of diluted samples, 50 μL of SA-d4 (5 mM) were added, and 2.5–5 μL were injected in the LC–MS/MS system. The enzyme activity was expressed after subtraction of the unspecific aspirin hydrolysis obtained by running in duplicate each sample of the study, omitting plasma addition.

### Pharmacokinetics study

For PK study, 6 mL of blood were collected in K-EDTA chilled tubes (3 mL) containing 20 μL of potassium fluoride 150 mg/mL (to minimize the hydrolysis of ASA to SA in human blood). The chilled blood samples were centrifuged immediately at 14,000*g* for 10 min at 4 °C, and the supernatant serum samples were frozen at − 20 °C until LC–MS/MS analysis.

Volunteers were randomly treated with EC-ASA or plain-ASA (100 mg/die) for  7 days before the study. On the 8th day, they fasted overnight and came to the laboratory at 09:00 a.m. after a light breakfast for the pharmacokinetics study. A blood sample was immediately collected at 9:00 a.m. (i.e. 24 h after the last dose intake) and then a 100 mg dose of EC-ASA or plain-ASA was administered. Blood was withdrawn at 2, 3, 4, 5, 6, and 8 h after EC-ASA, or at 0.5, 1, 1.5, 2, 3, 4, 5, and 6 h after plain-ASA administration.

After a wash-out period of almost 14 days, the aspirin formulations were switched, and the patients repeated the pharmacokinetic study. The wash-out period was necessary to allow the complete disappearance of residual effect (ASA irreversibly inhibit COX-1 in platelets, whose life-span is about 8–10 days).

### Quantification of thromboxane B_2_

For TxB_2_ quantification, 3 mL of blood were collected in tubes without anticoagulants (in order to obtain serum). Non-anticoagulated blood was allowed to clot in a water bath at 37 °C for 1 h. Then centrifuged at 1400*g* for 15 min, and serum samples were frozen at − 20 °C until analysis.

TxB_2_, the stable metabolite of TxA_2_, was measured by an enzyme immunoassay. Frozen samples were thawed at 37 °C and opportunely diluted (between 1:2 and 1:750) with PBS and tested in duplicate. Samples were assayed in parallel with the standard calibration curve (detection limit = 1.6 pg/mL), prepared as outlined in the manufacturer's instruction. The 96-well plate was read at 450 nm wavelength using Ensight multimode Reader. Results were expressed as ng/mL.

### Statistics

Statistical analyses were performed using GraphPad Prism v. 7.0 (GraphPad Software Inc, CA, USA). Peak integration and analytical method validation were performed using ABSciex Multiquant Software Version 2.0.

Results were expressed as either mean ± SD, mean ± SEM, or median and IQ range. To assess significance among the three studied groups one-way ANOVA or Kruskal–Wallis test were performed. Statistical significance was assumed at p < 0.05.

### Ethics approval

All procedures performed in studies involving human participants were in accordance with the ethical standards of the institutional and/or national research committee and with the 1964 Helsinki Declaration and its later amendments or comparable ethical standards.

### Consent to participate

Informed consent was obtained from all individual participants included in the study.

## Supplementary Information

Supplementary Information.

## Abbreviations

ASA: Aspirin
EC-ASA: Enteric-coated aspirin
HS: Healthy subjects
SA: Salicylic acid
PK: Pharmacokinetics
Plain-ASA: Plain aspirin
PD: Pharmacodynamics effect
