# Supplementary material for: In-vitro and in-vivo metabolism of different aspirin formulations studied by a validated liquid chromatography tandem mass spectrometry method
Source: Sci Rep. 2021 May 14;11:10370. doi: 10.1038/s41598-021-89671-w (PMC8121850; doi:10.1038/s41598-021-89671-w)
Supplement: Supplementary file 1 — Supplementary Information. [file 41598_2021_89671_MOESM1_ESM.docx]

**SUPPLEMENTAL DATA**

**In-vitro and in-vivo metabolism of different aspirin formulations studied by a validated liquid chromatography tandem mass spectrometry method**

Michele Dei Cas^a,•^, Jessica Rizzo^a,b,•^, Mariangela Scavone^b^ ,Eti Femia^b^, Gian Marco Podda ^b,c^, Elena Bossi^b^, Monica Bignotto^d^, Sabrina Caberlon^c^, Marco Cattaneo^b,c^ and Rita Paroni ^a,*^

^a^Laboratory of Clinical Chemistry and Mass Spectrometry, Department of Health Sciences, Università degli Studi di Milano, Milano, Italy

^b^Laboratory of Hemostasis and Thrombosis, Department of Health Science, Università degli Studi di Milano, Department of Health Science, Università degli Studi di Milano, Milano, Italy

^c^ Medicina III, ASST Santi Paolo e Carlo, Milano, Italy

^d^ lnternal Medicine and Liver Unit, Department of Health Sciences, Universita’ degli Studi di Milano, Milan, Italy

**Note S1. Optimization of mass spectrometry and liquid chromatography conditions**

For the detection and measurement of ASA and SA by Multiple Reaction Monitoring (MRM) tandem mass spectrometry, the most abundant precursor/product ion pair of each analyte and stable isotope-labelled analogue internal standard were selected (Figure S1) and the corresponding values of collision energy optimized to the best sensitivity.

In particular, the full Fragment ion spectra were recorded in infusion experiments, where also source parameters were optimized, as follows: ion source temperature 500 °C; ion spray voltage -4500V; nebulizing gas pressure 30 psi; heater gas pressure 45 psi, curtain gas pressure 45 psi and the collision energy was set at medium. For each analyte and IS, compound-dependent parameters: declustering potential (DP) entrance potential (EP), collision cell entrance potential (CEP) and collision cell exit potential (CXP) were optimized (Table S1). For MRM, the dwell time was set at 250 msec for each transition.

Figure S1 shows the integrated fragment spectra of ASA (above) and SA (below), from which the transitions m/z 179.0>137.0 for ASA and m/z 137.0>93.0 for SA were identified as the most suitable for detection. The corresponding transitions for the stable isotope-labelled internal standards are for ASA-d_4_ m/z 183.0>141.0 and for SA-d_4_ m/z 141.0>97.0.

For each transition, the optimal value of the collision energy was selected by recording and elaborating the fragment abundance curve. Fragment abundance curves were extracted from the instrument data file as .txt arrays of the fragment abundance *vs.* collision voltage from the Analyst software. All further elaboration was performed in custom Microsoft Excel spreadsheets, essentially according to Rubino *et al.* (Rubino et al., 2006, 2008).

As an example, Figure S2 shows the fragment abundance curve (an operational surrogate of the “breakdown curve”, adapted to the specific task) of the 137.0>93.0 transition of salicylic acid, elaborated to obtain the collision energy corresponding to the maximum yield of the selected fragment ion.

Rubino FM, Pitton M, Brambilla G, Colombi A. A study of the glutathione metaboloma peptides by energy-resolved mass spectrometry as a tool to investigate into the interference of toxic heavy metals with their metabolic processes. J Mass Spectrom. 2006 Dec;41(12):1578-93. doi: 10.1002/jms.1143. PMID: 17136764

Rubino FM, Pitton M, Caneva E, Pappini M, Colombi A. Thiol-disulfide redox equilibria of glutathione metaboloma compounds investigated by tandem mass spectrometry. Rapid Commun Mass Spectrom. 2008 Dec;22(23):3935-48. doi: 10.1002/rcm.3810. PMID: 19003853


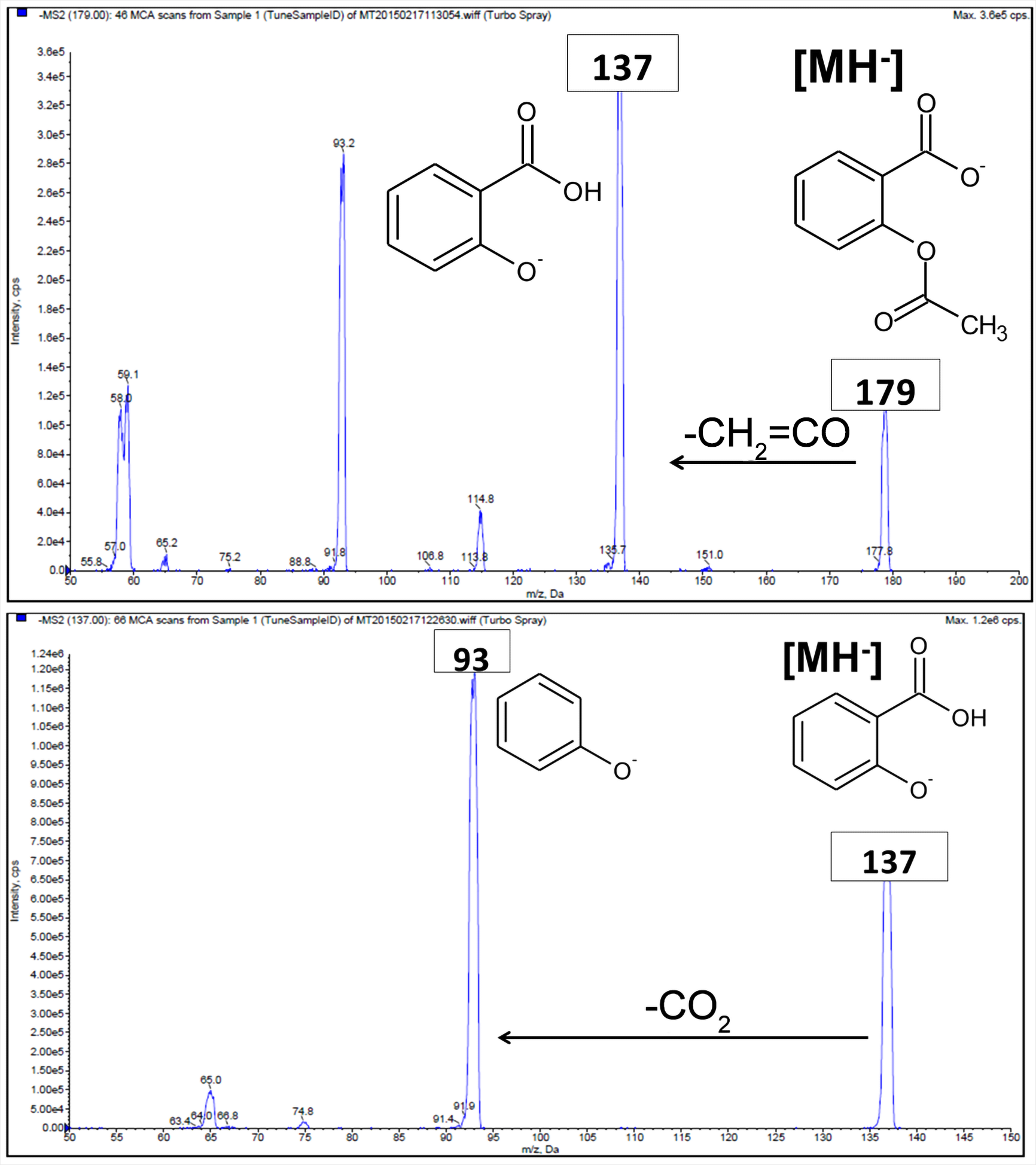


**Figure S1.** Fragment ion spectrum of ASA (above) and SA (below) electrosprayed in the negative ion mode. Precursor and fragment intensities are integrated over the -5 to -45 V interval of collision energies. The main ion species are annotated and the respective generation pathways are reported.


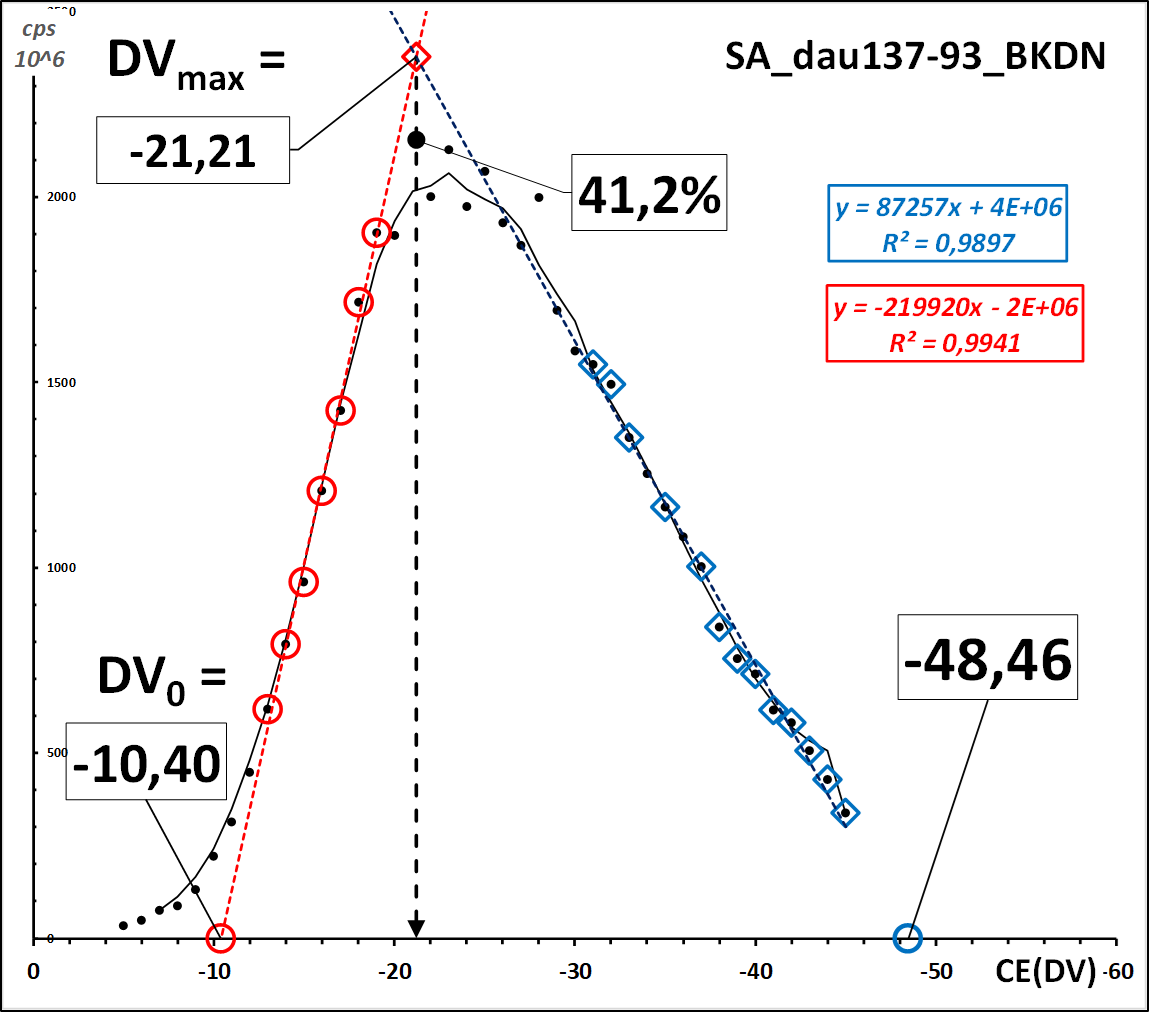


**Figure S2.** Breakdown curve of m/z 93 from m/z 137 in the MS-MS spectrum of electrosprayed salicylate. Reported are the values of the «laboratory-frame» collision energies (as collision cell voltage, in Volts) that correspond to the characteristic points of the curve. The maximum is appreciated with the «*teepee*» method, at the intersection of the lines that best-match the raising and descending intervals of the fragment intensity *vs.* collision energy curve. Reported also is the value of the percent fractional intensity of the fragment curve, referred to the total ion current in the selected collision energy range.

**Table S1.** Compound-specific operating parameters for each analyte and internal standard.

| Compound | Q1 (m/z) | Q3 (m/z) | DP (V) | EP (V) | CEP (V) | CE (V) | CXP(V) |
| --- | --- | --- | --- | --- | --- | --- | --- |
| ASA | 179 | 137 | -10 | -10 | -10 | -14 | -2 |
| ASA-d4 | 183 | 141 | -10 | -10 | -10 | -14 | -2 |
| SA | 137 | 93 | -33 | -10 | -10 | -25 | -2 |
| SA-d4 | 141 | 97 | -33 | -10 | -10 | -25 | -2 |


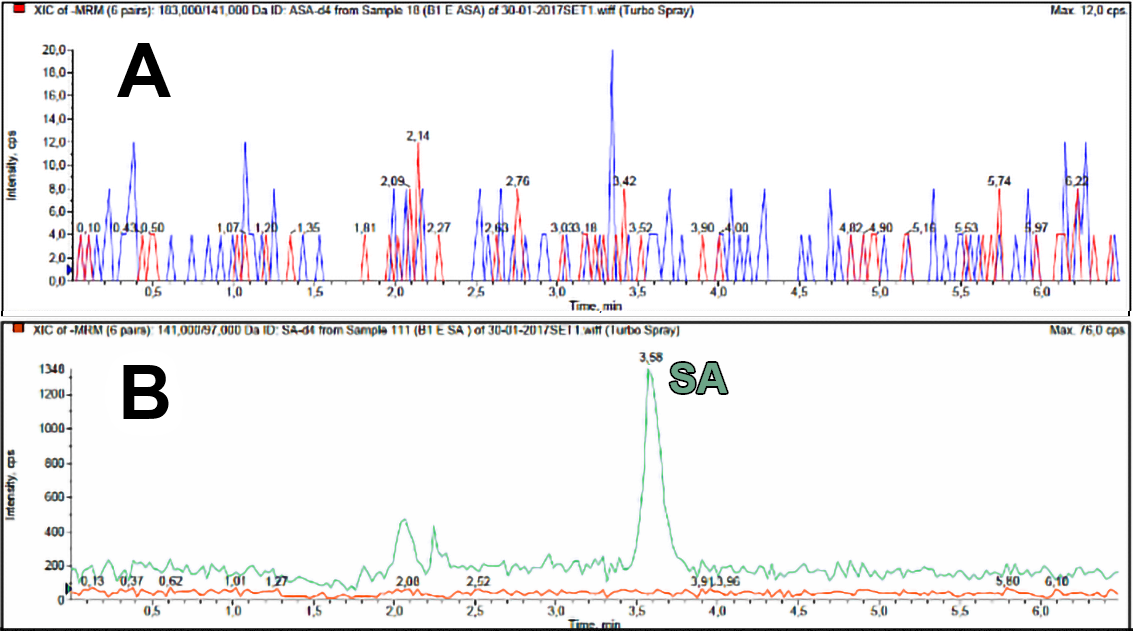


**Figure S3. (A)** Chromatograms of ASA (blue trace) and ASA-d4 (red trace) in human blank plasma and (**B**) chromatogram of SA (green trace) and SA-d4 (orange) in human blank plasma. In (**B**) it is displayed the SA peak, which corresponded to its physiological level.


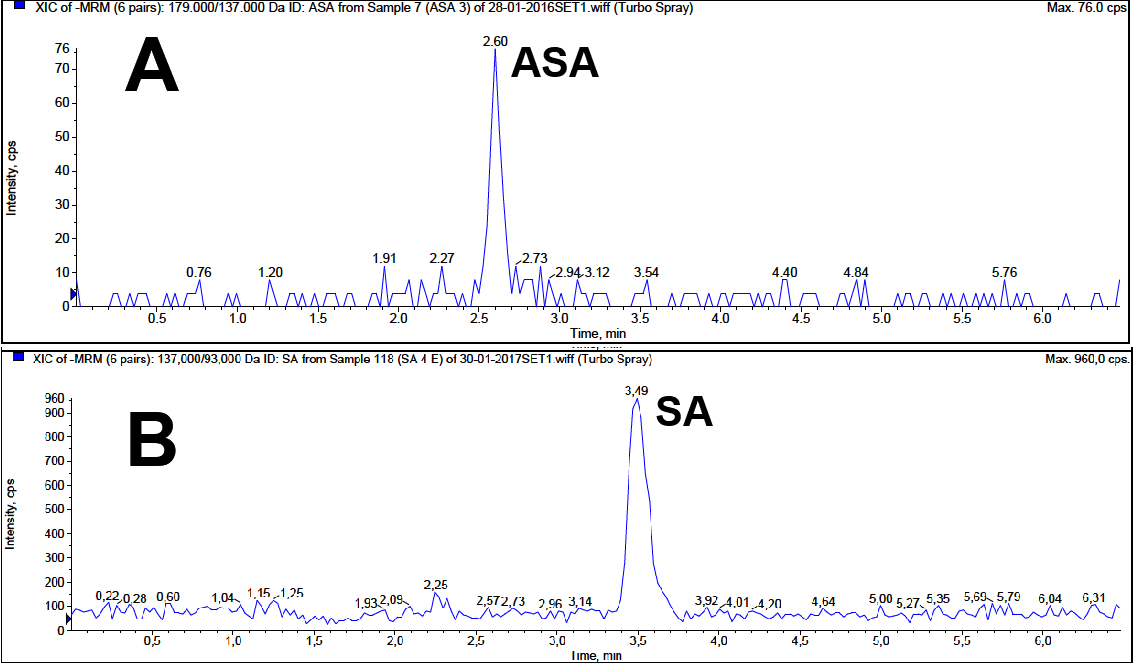


**Figure S4.** LLOQs chromatograms of ASA (**A**, 20 ng/mL) and SA (**B**, 20 ng/mL).

**Table S2**. Intra-day precision and accuracy for ASA and SA QCs in human plasma (n=6). Concentration found is expressed as mean±SD.

| Analyte | Nominal concentration (ng/mL) | Concentration found (ng/mL) | Accuracy (%) | Precision (%) |
| --- | --- | --- | --- | --- |
| ASA | 20 | 18.8±3.11 | 93.8 | 16.6 |
|  | 60 | 52.3±3.63 | 87.0 | 6.94 |
|  | 400 | 433±18.1 | 108.2 | 4.18 |
|  | 1250 | 1230±28.6 | 98.4 | 2.33 |
| SA | 20 | 23.8±3.07 | 119.0 | 12.9 |
|  | 60 | 66.0±4.03 | 110.0 | 6.10 |
|  | 200 | 225±4.09 | 112.7 | 1.81 |
|  | 500 | 561±21.9 | 112.2 | 3.91 |
|  | 5000 | 5770±55.4 | 115.4 | 0.96 |

**Table S3.** Inter-day precision and accuracy for ASA and SA QCs in human plasma (n=6). Concentration found is expressed as mean±SD.

| Analyte | Nominal concentration (ng/mL) | Concentration found (ng/mL) | Accuracy (%) | Precision (%) |
| --- | --- | --- | --- | --- |
| ASA | 20 | 18.2±2.23 | 91.1 | 12.2 |
|  | 60 | 52.2±3.75 | 87.0 | 7.17 |
|  | 400 | 385.2±39.8 | 96.3 | 10.3 |
|  | 1250 | 1214±72.4 | 97.2 | 5.96 |
| SA | 20 | 23.3±2.01 | 116 | 8.64 |
|  | 60 | 67.3±3.62 | 112 | 5.38 |
|  | 200 | 225±3.34 | 113 | 1.48 |
|  | 500 | 557±15.88 | 112 | 2.85 |
|  | 5000 | 5719±80.6 | 114 | 1.41 |

**Table S4.** Stability study for ASA QC in human plasma in the low (60 ng/mL) and high (1250 ng/mL) range. Concentrations are reported as mean±SD of 6 replicates

| Stability test | QC nominal concentration (ng/mL) | Concentration found (ng/mL) | Stability (%) | Precision (%CV) |
| --- | --- | --- | --- | --- |
| Autosampler stability (at 5°C for 72 h) | 60 (low) | 51.0±2.35 | 85.0 | 4.62 |
|  | 1250 (high) | 1136±60.3 | 90.8 | 5.31 |
| Short-term stability  (in ice-bath for 6 h) | 60 | 53.3±4.79 | 88.8 | 8.99 |
|  | 1250 | 1247±107 | 99.8 | 8.56 |
| Long-term stability  (at -20°C for 60 day) | 60 | 51.7±1.29 | 86.1 | 2.49 |
|  | 1250 | 1258±94.4 | 100 | 7.50 |
| Freeze-thaw stability (after 3 cycles) | 60 | 50.1±0.67 | 83.5 | 1.35 |
|  | 1250 | 1109±98 | 88.7 | 8.80 |

**Table S5.** Stability study for SA QC in human plasma in the low (60 ng/mL), medium (200 ng/mL), and high (5000 ng/mL) range. Concentrations are reported as mean±SD of 6 replicates

| Stability test | QC nominal concentration (ng/mL) | Concentration found (ng/mL) | Stability (%) | Precision (%CV) |
| --- | --- | --- | --- | --- |
| Autosampler stability (at 5°C for 72 h) | 60 (low) | 68.9±0.05 | 114.8 | 0.07 |
|  | 200 (medium) | 225±0.29 | 112.6 | 0.13 |
|  | 5000 (high) | 5713 ±49.9 | 114.2 | 0.87 |
| Short-term stability (in ice-bath for 6 h) | 60 | 68.9±1.89 | 114.9 | 2.75 |
|  | 200 | 198.5±6.95 | 99.25 | 3.50 |
|  | 5000 | 5058±236 | 101.2 | 4.66 |
| Long-term stability (at -20°C for 60 day) | 60 | 68.5±3.16 | 114.2 | 4.61 |
|  | 200 | 223.2±4.31 | 111.6 | 1.93 |
|  | 5000 | 5747±46.2 | 115.0 | 0.80 |
| Freeze-thaw stability (after 3 cycles) | 60 | 68.9±2.81 | 114.8 | 4.09 |
|  | 200 | 226±2.91 | 112.8 | 1.29 |
|  | 5000 | 5743±35.7 | 114.9 | 0.62 |
